# Assessing the accuracy and efficacy of multiscale computational methods in predicting reaction mechanisms and kinetics of S_N_2 reactions and Claisen rearrangement

**DOI:** 10.1038/s41598-024-67468-x

**Published:** 2024-07-22

**Authors:** Maryam Haji Dehabadi, Hamid Saidi, Faezeh Zafari, Mehdi Irani

**Affiliations:** Department of Chemistry, University of Kurdistan, Sanandaj, 66177-15175 Iran

**Keywords:** Multiscale modeling, ORCA software, QM/MM, Reaction mechanism, Python scripting, Organic chemistry, Theoretical chemistry

## Abstract

This study investigates the application of quantum mechanical (QM) and multiscale computational methods in understanding the reaction mechanisms and kinetics of S_N_2 reactions involving methyl iodide with NH_2_OH and NH_2_O^−^, as well as the Claisen rearrangement of 8-(vinyloxy)dec-9-enoate. Our aim is to evaluate the accuracy and effectiveness of these methods in predicting experimental outcomes for these organic reactions. We achieve this by employing QM-only calculations and several hybrids of QM and molecular mechanics (MM) methods, namely QM/MM, QM1/QM2, and QM1/QM2/MM methodologies. For the S_N_2 reactions, our results demonstrate the importance of explicitly including solvent effects in the calculations to accurately reproduce the transition state geometry and energetics. The multiscale methods, particularly QM/MM and QM1/QM2, show promising performance in predicting activation energies. Moreover, we observe that the size of the MM active region significantly affects the accuracy of calculated activation energies, highlighting the need for careful consideration during the setup of multiscale calculations. In the case of the Claisen rearrangement, both QM-only and multiscale methods successfully reproduce the proposed reaction mechanism. However, the activation free energies calculated using a continuum solvation model, based on single-point calculations of QM-only structures, fail to account for solvent effects. On the other hand, multiscale methods more accurately capture the impact of solvents on activation free energies, with systematic error correction enhancing the accuracy of the results. Furthermore, we introduce a Python code for setting up multiscale calculations with ORCA, which is available on GitHub at https://github.com/iranimehdi/pdbtoORCA.

## Introduction

### Major classes of computational chemistry methods

Computational chemistry comprises two main classes: quantum mechanical (QM) and classical molecular mechanics (MM) methods. QM methods utilize a Hamiltonian expression for the treatment of electrons, enabling them to predict properties such as bond energies, charge distributions, and reaction pathways with high accuracy. Additionally, QM methods offer promising avenues for exploring reaction mechanisms. Conversely, MM methods employ classical physics to model molecules, treating atoms as point masses and point charges, and bonds as springs, thereby ignoring the electronic structure and cannot accurately simulate bond breakings and formation. Therefore, MM methods are suitable for studying the overall structure, conformational changes, and dynamics of molecules.

A notable distinction between these methods lies in computational efficiency, particularly in terms of computation time. While MM methods offer significantly faster calculations, QM methods, such as density functional theory (DFT) are much slower. It is important to note that the computational time of DFT scales proportionally to N^4^, where N represents the size of the system.^1^ Consequently, this imposes limitations on the number of atoms that can be feasibly included in DFT calculations (typically around 200 atoms). Moreover, QM methods struggle to accurately simulate organic reactions in solution phases due to the complexity arising from the extensive solvent molecules surrounding organic molecules. Attempting to model such extensive systems solely with QM methods becomes infeasible from a hardware perspective.

To tackle the complexities associated with the modeling of the reaction environment in an organic reaction, two primary strategies emerge, implicit or explicit modeling of the solvent medium. In the former, the solvent is modeled implicitly as a continuum environment with a specific dielectric constant as that of the solvent such as the conducting polarizable continuum model (CPCM).^2^ This presents a computationally less demanding method. On the other hand, in the latter, the reaction medium is considered explicitly, representing the reaction environment through methods such as semiempirical or MM. While offering a more detailed representation of the reaction environment, this method incurs a higher computational cost due to its explicit modeling approach. A notable challenge with explicit modeling lies in the complexity of handling the QM and MM regions, which can lead to intricacies and potential errors for the user. However, computer programs can aid in navigating these complexities, offering solutions for the setup.

### Multiscale implementation in *ORCA*

Some QM programs such as Gaussian,^3^ Psi4,^4^ and ORCA,^5^ alongside semiempirical programs like Mopac^6^ and DFTB + ,^7^ have been developed to facilitate hybrid QM/MM calculations. Among these, ORCA stands out as an efficient and user-friendly software solution for quantum chemistry, offering versatility across various computational tasks. In this work, we utilized the ORCA program to perform multiscale calculations. Further details about the program can be found in the Supporting Information.

Within ORCA, three distinctive multiscale implementations stand out: QM/MM, QM1/QM2, and QM1/QM2/MM. The QM/MM approach seamlessly integrates QM calculations to elucidate the electronic structure of a specified region within a system, such as the active site in enzymatic reactions, complemented by MM to model the remainder of the system. However, QM1/QM2 employs two QM levels (QM1 and QM2) to describe different facets of the system. QM1 ensures a high-precision representation of the reaction active region, while QM2 captures the surroundings at a lower computational cost without compromising accuracy. Building upon this, QM1/QM2/MM introduces MM as a third level of description. This advancement allows for a more intricate and accurate depiction of the system by seamlessly incorporating MM to represent the effects of the surrounding environment, while harnessing the precision of QM calculations in the reaction active region. These multiscale implementations offer a spectrum of approaches, providing researchers with the flexibility to tailor computational strategies to align with specific research objectives and the available computational resources.

**Figure 1:**
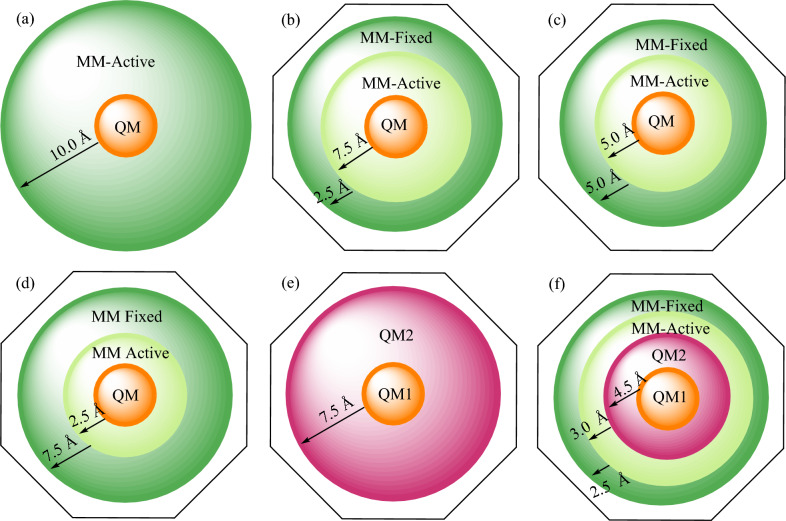
Schematic representations depict the QM, QM1, QM2, MM-active, and MM-fixed regions, along with the excluded portion of the system (shown in white) in various types of multiscale calculations conducted using ORCA. Throughout this study, we ensured consistent QM or QM1 regions across all calculations while adjusting the thicknesses of the QM2, MM-active, and MM-fixed regions, as illustrated. The numbers show the thicknesses for each region in Å.

In QM/MM calculations facilitated by ORCA, the QM region can be specified either directly by the user within an ORCA input file (e.g., using the syntax '*QMAtoms {1 2 27 28} end* ' in the *%qmmm* block) or through flags in a PDB file describing the system (indicated by the value 1.00 in the occupancy column). Additionally, the MM region comprises atoms situated within a sphere around the QM region and is treated by an MM method. These two regions collectively form active regions. The atoms within the active region can be defined either directly within an ORCA input file (e.g., using '*ActiveAtoms {1 2 27 28 30 32:40} end* ' in the *%qmmm* block) or via the B-factor column of a PDB file (indicated by 1.00 in the B-factor column). A sample ORCA input file utilized in this study is compiled in the Supporting Information. Additionally, the QM and MM-active regions (referred to as the active region) are visually depicted in orange and light green, respectively, in Fig. 1.

In ORCA's multiscale geometry optimization, the optimization process only affects the positions of the active atoms, while the forces on these atoms are influenced by interactions with non-active surrounding atoms. To ensure smooth optimization convergence for quasi-Newton optimization algorithms in internal coordinates, the Hessian values between the active atoms and the directly surrounding non-active atoms must be available. For this purpose, the active atoms are extended by a shell of surrounding non-active atoms, which are also included in the geometry optimization but have their positions constrained. This region is referred to as the MM-fixed or extension shell, depicted in dark green in Fig. 1. ORCA provides three methods for automatically determining the extension shell: *Distance*, where non-active atoms within a specified distance from active atoms are included; *Covalent*, which bonds all non-active atoms covalently bonded to active atoms; and *No*, where no non-active atoms are included. Furthermore, users have the flexibility to manually specify atoms for the extension shell according to their specific requirements, utilizing the *OptRegion_FixedAtoms* keyword in the %*qmmm* block in the input file.

In QM1/QM2/MM calculations, the treatment of the MM region remains consistent with QM/MM calculations, however, users have the option to define a second QM region surrounding the primary QM region, as illustrated in Fig. 1f. Conversely, in QM1/QM2 calculations, the MM region is absent, and the system comprises two distinct QM regions, as depicted in Fig. 1e.

### Computational methods and reaction kinetics and mechanism

Experimental exploration of reaction mechanisms and kinetics necessitates the identification of intermediates and transition states (TSs) along the reaction pathway, along with understanding the energetic profiles of each reaction step. However, intermediates and particularly TSs are often fleeting and challenging to characterize experimentally. Specialized experimental techniques may be employed to capture and analyze these short-lived chemical species, necessitating costly instrumentation and hazardous chemicals. In contrast, computational chemistry methods offer a more viable approach. These methods enable the characterization and optimization of geometrical structures of stationary structures along a reaction path, allowing for the determination of the energy associated with each optimized state. Crucially, computational methods circumvent the need to physically trap short-lived intermediates and TSs, offering a more efficient alternative to experimental approaches. Moreover, these computational techniques solely consume electrical energy, avoiding the use of hazardous chemicals associated with experimental methods.

One of the key advantages of computational methods lies in their capability to calculate activation energies for reactions, providing valuable insights that experimentalists can utilize to gauge the feasibility of certain reactions. By identifying reactions deemed infeasible through computational modeling, experimentalists can forego conducting these reactions in the laboratory, thereby saving resources and mitigating potential risks. In computational chemistry, multiscale methods offer a more feasible approach to model chemical reactions and study their kinetics. Given that most reactions occur in solution phases, which significantly influence reaction dynamics, particularly in organic chemistry, it becomes crucial to account for solvent effects. Both explicitly and implicitly, solvents play a pivotal role in chemical reactions. Therefore, to accurately model the solvent environment, multiscale methods such as QM/MM are necessary. These methods allow for a comprehensive representation of the solvent medium, enabling a more accurate simulation of chemical reactions within their natural environment.

### The studied reactions

**Figure 2:**
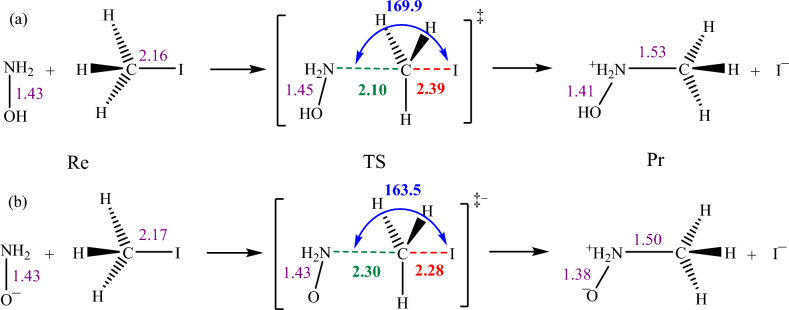
The reaction between CH_3_I and (**a**) NH_2_OH and (**b**) NH_2_O^−^. The figure presents the average key geometrical parameters obtained from the multiscale methods employed in studying the S_N_2 reactions. Distances are expressed in Å, and the NCI angles are in degrees. The average distances are shown in three colors: green for forming bonds, red for breaking bonds, and purple for bonds that remain unchanged. The NCI angle is shown in blue. The complete set of geometrical parameters is provided in Tables S1 and S2 in the Supporting Information. Notably, nucleophilic attack consistently occurs via the nitrogen atom of the nucleophiles rather than the oxygen atom.

In this work, using the multiscale methods, we investigate bimolecular nucleophilic substitution (S_N_2) reactions involving methyl iodide (CH_3_I) and either NH_2_OH or NH_2_O^−^ as depicted in Fig. 2. These reactions follow second-order kinetics, indicating that their rate is dependent on the concentrations of both reactants. During the process, the nucleophile (NH_2_OH or NH_2_O^−^) attacks the substrate (CH_3_I) from the opposite side of the leaving group (I^−^). This interaction triggers the creation of a new C−N bond while simultaneously breaking the existing C−I bond and releasing an iodide ion. An essential feature of these reactions is the inversion of configuration at the electrophilic carbon of the substrate. This inversion is a crucial aspect of the S_N_2 mechanism wherein the nucleophile replaces the leaving group, resulting in a product with a configuration opposite to that of the initial reactants. Notably, nucleophilic attack consistently occurs via the nitrogen atom of the nucleophile rather than its oxygen atom. Our calculations confirmed this, showing that the nucleophilic attack through oxygen is energetically very high, prohibiting the reaction.

Also, we study the Claisen rearrangement of sodium 8-(vinyloxy)dec-9-enoate (cf. Fig. 2) in the mixture of water/methanol solvents with different weight percentages; 100/0%, 75/25%, 50/50%, 25/75%, and 0/100%. This rearrangement is a valuable tool in organic synthesis for constructing carbon–carbon bonds and forming complex molecular structures. It is widely used in the synthesis of natural products and pharmaceuticals. The reaction proceeds through a highly ordered cyclic transition state and is intramolecular.

For the S_N_2 reactions, experimental activation energies are available in an aqueous solution^8^, while for the Claisen rearrangement, experimental kinetics data are available in water, methanol, and mixtures of water and methanol^9^. The two S_N_2 reactions share similarities but differ in the charge of the nucleophile: one is neutral (NH_2_OH), and the other is negatively charged (NH_2_O^−^). The negatively charged nature of NH_2_O^−^ enhances its nucleophilicity, leading to a decrease in activation energy, as reflected in the experimental data (from 23.5 to 16.1 kcal/mol)^8^. Additionally, studies by White^10^ and the Cornell^11^ research group have shown that polar solvents enhance the rate of Claisen rearrangements. To explore solvent effects, we investigated the kinetic parameters of the two S_N_2 reactions in aqueous solution and the Claisen rearrangement in mixtures of water/methanol solvents with varying weight percentages, reflecting different polarities. By employing various variants of multiscale methods and comparing the activation energies obtained from them, we aim to identify the most effective method for reproducing experimental the observations.

### Aims

**Figure 3:**
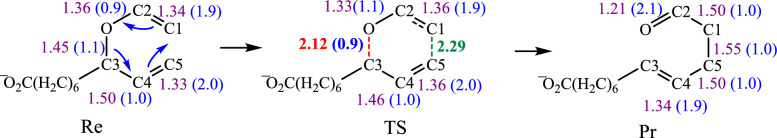
Claisen rearrangement of sodium 8-(vinyloxy)dec-9-enoate. The figure presents the average of the distances on the reaction center (in Å) obtained from both QM-only and multiscale methods employed in studying this reaction. The complete set of geometrical parameters is provided in Tables S3-S8 in the Supporting Information. The average distances are shown in three colors: green for forming bonds, red for breaking bonds, and purple for bonds that remain unchanged. Additionally, Mayer bond orders for each bond from the QM-only calculations are shown in blue.

Despite some computational studies have been conducted on these or related reactions^12–19^, there is currently no report comparing the efficiency of different computational methods in predicting their mechanisms and kinetic parameters. In this study, we aim to assess the effectiveness of QM-only, QM/MM, QM1/QM2, and QM1/QM2/MM methodologies in replicating the generally accepted reaction mechanisms and experimentally reported kinetic parameters for reactions involving methyl iodide with NH_2_OH and NH_2_O^−^ (Fig. 2) and the Claisen rearrangement of the vinyloxy compound (Fig. 3). To achieve this objective, we employ various variants of these multiscale methods, adjusting the thickness of the MM-active, MM-fixed, and QM2 regions in the calculations, as illustrated in Fig. 1. Through comparative analyses, our goal is to identify the superior method among these approaches for accurately reproducing the experimental kinetics parameters. Additionally, we provide a Python code to facilitate setting up multiscale calculations for ORCA, addressing the challenges associated with manually assigning the MM-free, MM-fixed, and QM2 regions.

## Methods

### QM calculations

The QM calculations were conducted using the ORCA program package (version 5.0.3), which includes support for libXC version 5.1.0^20,21^. The TPSSH functional^22^ and the def2-SVP basis set^23^ were employed for these calculations. The auxiliary def2/J basis set^24^ was utilized to enhance computational efficiency, and atom-pairwise dispersion correction was applied using the Becke-Johnson damping scheme (D3BJ)^25,26^.

The choice of the TPSSH functional for our calculations stems from its demonstrated performance in recent chemical studies^27,28^. Known for delivering dependable geometries and offering computational efficiency, this functional strikes a balance between accuracy and computational cost. Its efficacy in capturing the nuances of chemical systems makes it a fitting choice for our investigation into reaction mechanisms and kinetics.

In some instances, the QM systems were immersed within a continuum solvent using the CPCM model^2^. For this, the stationary structures were first optimized in vacuum with the QM-only method, and then the continuum model calculations were performed as single-point calculations on the optimized structures. The default optimized CPCM parameters were used to generate solvent-accessible surface cavities for pure water and pure methanol solvents. For solvent mixtures of water and methanol, dielectric constants were obtained from an experimental study^29^. Although dielectric data for all mixtures were not available, a linear correlation between the mass percentage of methanol and dielectric constants was observed, with an *R*^2^ value of 0.99 (refer to Figure S1 in the Supporting Information). Leveraging this experimental correlation^29^, dielectric constants for methanol/water solvents with weight percentages of 25/75%, 50/50%, and 75/25% were determined as 56.033, 45.28, and 35.215, respectively.

The choice of the CPCM solvation model was primarily driven by our aim to compare calculated activation energies or activation free energies from the simple, less demanding implicit solvation models with those from complex, high-demanding explicit solvation models. No specific preference was given to CPCM over other implicit solvation models. Additionally, CPCM requires fewer parameters. This feature proved particularly useful for our study involving mixtures of methanol and water solvents, as CPCM enabled us to input missing parameters for these solvent mixtures directly into the ORCA input files, thereby providing a distinct advantage over other implicit solvation models.

### Multiscale calculations

#### System equilibration

To initiate multiscale calculations, equilibrated structures of the systems under study, a topology file, and an ORCA input file delineating the system's different regions (cf. Fig. 1) are required. We generated the equilibrated PDB and topology files using the AMBER program package^30^. The TIP3P water model^31^ was employed to characterize water molecules, while the reactants and methanol were parameterized using the general AMBER force field (GAFF)^30^. To obtain the GAFF parameters, initially, we optimized the geometric structures of the reactants or the solvents at the B3LYP/6-31G(d) level of theory^32–36.^ Due to the unavailability of the 6-31G(d) basis set for iodine, the LANL2DZ^37^ basis set was employed for the iodine atom in methyl iodide. Additionally, the Merz-Kollman atomic radius for iodine was set at 1.98 Å. Subsequently, electrostatic potentials were computed at the Hartree − Fock/6-31G(d) level of theory, employing points sampled according to the Merz − Kollman scheme^38^ via the Gaussian 16 program package^39^. Atomic charges were derived from these potentials using the RESP procedure^40^, implemented in the antechamber module of the AMBER program^40^, which also assigned GAFF atom types to the molecules. Detailed GAFF parameters are provided in the Supporting Information.

To simulate reactions in pure water, we solvated the reactants in an octahedral system using TIP3P water molecules^31^, ensuring that solvent molecules extended at least 20 Å from the reactants. Subsequently, the system underwent energy minimization over 1000 cycles, followed by equilibration for 10 ps at constant volume. Finally, the systems underwent equilibration through a 1 ns constant-volume simulation and a 1 ns simulated annealing at constant pressure, restraining the reactant structure while allowing the solvent to fully relax (the force constant for the restraints in all steps was 1000 kcal/mol/). Bond lengths involving hydrogen atoms were constrained using the SHAKE algorithm^41^, enabling a time step of 2 fs during the simulations. The temperature was maintained at a constant 300 K using Langevin dynamics^42^ with a collision frequency of 2 ps^–1^. The pressure was held constant at 1 atm utilizing Berendsen’s weak coupling isotropic algorithm^43^ with a relaxation time of 1 ps. Long-range electrostatics were managed via particle-mesh Ewald summation^44^ with a fourth-order B-spline interpolation and a tolerance of 10^–5^. The cut-off radius for Lennard‒Jones interactions was set to 8 Å.

The octahedral shape of the solvent sphere is preferred due to its ability to minimize the number of solvent molecules required to surround the solute, thus reducing computational costs. As far as we know, the octahedral shape is only available for the pure water solvent in AMBER. Hence, to model the Claisen rearrangement of the vinyloxy in pure methanol and methanol/water solvent mixtures with different weight percentages (100/0%, 25/75%, 50/50%, 75/25%), we utilized the Packmol package^45,46^. The number of the first solvent molecules (*N*1) in the mixture was determined using Eq. 1:

1 $$N{1}\, = \,\left( {W{1}\% \, \times \,a^{{3}} \, \times \,d\, \times \,N_{{\text{A}}} } \right) \, /M{1})\, \times \,{1}0^{{ - {26}}}$$

where *W*1% represents the mass percent of the first solvent (note that the 10^−26^ factor in the equation should be 10^−24^ if you use the mass fraction, a number between 0 and 1, instead of mass percentage), *a* is the length of the cubic box in Å, *d* is the density of the solvent mixture expressed in g/cm^3^, *N*_A_ is Avogadro's number, and *M*1 is the molar mass of the first solvent given in g/mol. A similar was used to obtain the number of the second solvent molecules in the mixtures. Density data for the mixtures were obtained from an experimental study^47^. A linear correlation between the mass percentage of methanol and density was observed in the experimental data, with an *R*^2^ value of 0.99 (refer to Figure S2 in the Supporting Information).

After determining the number of solvent molecules, we solvated the reactants in a cubic box with dimensions of 30 Å × 30 Å × 30 Å using the Packmol package^45,46^ (a Packmol input file is prepared in the Supporting Information). The systems were then equilibrated using the AMBER program^30^. Due to the systems' departure from an equilibrium state from the Packmol package, energy minimization was conducted over 1000 cycles. Subsequently, the system's temperature was gradually and slowly raised from 0 to 300 K in six heating stages over 30 ps each (with 10,000 iterations and a time step of 0.05 fs). Finally, the systems were equilibrated at 300 K in two stages: firstly, through a 1 ns simulation with position restraints on heavy atoms with a force constant of 0.1 kcal/mol/Å^2^, and secondly, through a 1 ns simulation allowing heavy atoms to move freely with a time step of 2 fs. During these steps bond lengths involving hydrogen atoms were constrained using the SHAKE algorithm^26^.

Periodic Boundary Conditions (PBC) were applied for the octahedral systems to ensure that the simulated system behaves as an infinite system, effectively avoiding edge effects. However, since the cubic systems were generated using the Packmol package, which does not provide cell parameters, we initially attempted to manually insert cell parameters into the coordinate files and run simulations with PBC. These simulations, however, resulted in significant errors, making the results unreliable. Consequently, we decided not to use PBC for the cubic systems.

#### Multiscale system setup and the *Python* code

Following the equilibration of solvated reaction systems, multiscale calculations were set up according to Fig. 1, incorporating varying sizes of the QM2, MM-active, and MM-fixed systems (with the central QM system consistently representing the reactants). To facilitate this setup, we developed a Python code, named "*pdbtoorca*," available on GitHub at https://github.com/iranimehdi/pdbtoORCA. This code generates ORCA input files (.*inp*) describing QM/MM calculations, requiring a PDB file and a file describing the QM system (default name: *s1*) as inputs. The thickness of the MM-active region around the QM system can be specified (default value: 6.0 Å), and the user can choose from four options to define the MM-fixed region. Additionally, the code prompts for the desired level of theory, the number of CPU cores for parallel calculations, and the charge and multiplicity of the QM region. Similarly, the program sets up QM1/QM2 calculations. For this, the user provides a PDB file of the system and the *s1* file, with options to specify the thickness and the level of theory of the QM2 region; defauls are 3.0 Å and XTB^48,49^, respectively. The charge and multiplicity of the medium (QM1 + QM2) system can also be assigned by the user, along with other parameters such as the number of CPU cores. Similarly, to generate input files for QM1/QM2/MM calculations, the user specifies the thickness of the QM2, MM-active and MM-fixed regions. Afterward, the ORCA input file is prepared to execute the calculations. Notably, in all QM1/QM2 and QM1/QM2/MM calculations, the QM2 region was treated using the semi-empirical XTB method as the chosen level of theory.

In ORCA software, users have the flexibility to choose between electrostatic or mechanical embedding for the interaction between QM and MM regions. Additionally, ORCA employs the additive scheme for QM/MM calculations and the subtractive scheme for QM1/QM2 and QM1/QM2/MM multiscale calculations. If the embedding type is not explicitly specified in the input file, the program defaults to electrostatic embedding. Importantly, ORCA recalculates the charges for the QM region during the geometrical optimization process, ensuring that the charges in the QM region, are dynamically updated based on the quantum mechanical environment. This dynamic updating of charges is critical for accurately modeling reactivity and is an integral part of our computational protocol.

We conducted multiscale calculations using both electrostatic and mechanical embedding. For the S_N_2 reactions, we initially employed electrostatic embedding to optimize the structures. Subsequently, mechanical embedding was utilized for single-point calculations on these electrostatically embedded structures. However, in the case of the Claisen rearrangement, we applied mechanical embedding either as single-point calculations on structures obtained through electrostatic embedding or by optimizing the structures directly with mechanical embedding. This dual application was chosen to test more variants of the multiscale methods for the Claisen rearrangement. For more comprehensive insights into embedding types and energy calculation methods in multiscale calculations, readers can refer to a review by Lily Cao and Ulf Ryde^50^.

To locate first-order stationary states (reactants and products), we optimized the structures using the corresponding multiscale method without any restraints. However, to locate transition states, we scanned reaction coordinates between reactants and products in steps of 0.2 Å (0.1 Å around the maximum point of energy). During the scan, all other degrees of freedom were allowed to relax. The TSs were then approximated as the highest point on the potential energy surface along the reaction coordinates. We confirmed that once the restraints were released, the TSs returned to their respective reactant or product states.

### Quality measures

Each method underwent evaluation based on several quality measures viz., the correlation coefficient (*R*^2^), relative range of the method with respect to the corresponding experimental range, mean signed error (MSE), mean absolute deviation (MAD), MAD after systematic error removal (MADtr), and maximum error (after systematic error removal; MAXtr), all relative to the experimental activation energy (*E*_a_) for the S_N_2 reactions or Δ*G*^‡^ for the Claisen rearrangement of the vinyloxy compound. Additionally, Spearman's rank correlation coefficient (ρ) and Kendall's τ were calculated to assess the accuracy of different methods in determining the correct order of *E*_a_ and Δ*G*^‡^.

It is important to note that the quality measures can be grouped into two categories, often exhibiting opposing trends. Metrics like *R*^2^, ρ, and τ indicate how effectively the various methods rank the different experimental values. *R*^2^, in particular, is sensitive to both the largest and smallest energy values, and all three tend to favor methods that overestimate the magnitude of the energies. Conversely, metrics such as range, MAD, MADtr, and MAXtr focus on the absolute values of the energies, penalizing methods that yield excessively large magnitudes and differences between the various energies. These measures may yield favorable results for methods that underestimate the energies. Thus, incorporating quality measures from both categories is crucial for deriving a comprehensive final score.

## Results and discussion

In the following, we embark on an exploration of the results obtained from our computational investigations. We begin by unraveling the intricate details of the mechanism underlying the S_N_2 reactions, shedding light on the activation energies extracted through the multiscale calculations. Next, we dissect the mechanism governing the Claisen rearrangement, analyzing the activation-free energy associated with this pivotal rearrangement process as unveiled by our multiscale computational calculations.

### Reaction mechanism of the studied S_N_2 reactions

In the generally accepted mechanism of S_N_2 reactions depicted in Fig. 2, the hallmark feature is the five-coordinated carbon atom with an approximately 180° angle between the attacking nucleophile, the substrate's carbon atom, and the leaving group in the TS structure. However, our results revealed that scanning the reaction path in vacuum did not reproduce this expected TS structure. Instead, it resulted in atypical angles and an incongruous structure. Notably, including the solvent implicitly using the CPCM model during both optimization and scan calculations did not significantly alter these observations. To successfully obtain the TS structure in vacuum, we had to constrain the NCI angle to ~ 180° during the scan process.

Given that vacuum calculations (QM-only calculations) do not faithfully reproduce the S_N_2 reaction mechanism, we conducted these reactions explicitly accounting for the solvent environment using the multiscale methods. We employed variants of the QM/MM method, i.e. using different thicknesses of the MM-active region around the QM region and MM-fixed region around the MM-active region (i.e., 10.0/0.0, 7.5/2.5, 5.0/5.0, 2.5/7.5 Å; the value preceding the slash indicates the thickness of the MM-active region, while the value following the slash denotes the thickness of the MM-fixed region), QM1/QM2 calculations with QM2 region thicknesses of 4.5 and 7.5 Å around the QM1 region, and QM1/QM2/MM calculations with QM2, MM-active, and MM-fixed regions sized at 4.5, 3.0, and 2.5 Å, respectively (cf. Fig. 1).

Following the multiscale calculations, we extracted geometrical data between heavy atoms (O–N, N–C, and C–I distances, and the NCI angle) of the reactant (Re), TS, and product (Pr) states for both S_N_2 reactions and collected them in Tables S1 and S2 in the Supporting Information. However, the average values of key geometrical parameters over the multiscale methods are presented in Fig. 2. Notably, the NCI angle approximates 180° in all methods, with longer N–C distances in TS compared to Pr and longer C–I distances in TS compared to the Re states. These results corroborate the proposed S_N_2 mechanism obtained through multiscale methods, a feat unachievable in vacuum calculations without constraints on the NCI angle.

Further analysis of the geometrical data reveals subtle differences between the NH_2_OH and NH_2_O^−^ reactions. Specifically, the N–C distance in the TS of the NH_2_OH reaction is shorter than the corresponding average distance in the NH_2_O^−^ reaction (2.10 versus 2.30 Å), while the average C–I distance in the TS of the NH_2_OH reaction is longer than the corresponding value in the NH_2_O^−^ reaction (2.39 versus 2.28 Å).

It is worth noting that QM/MM calculations with a 2.5 Å radius for the MM-active region did not yield a product due to the confined space, preventing reactants from approaching for reaction, thus resulting in the exclusion of results from this variant in our dataset.

### The activation energy of the S_N_2 reactions

Following the discussion of the S_N_2 reaction mechanism in the previous section, we now delve into the results obtained from multiscale calculations concerning the activation energy of these reactions. We excluded the QM-only results and the energies from the CPCM calculations because they do not yield the expected TS structure without angle constraints. This examination aims to determine which method better replicates experimental activation energies. To this end, we expanded the multiscale calculations by employing mechanical embedding as single-point calculations on stationary structures obtained from the corresponding calculations with electrostatic embedding.

Table 1 presents a comparison of the calculated activation energies from different theoretical methods with experimental values for these reactions.^8^ The table includes MAD, MADtr, MAXtr, relative range with respect to the experimental range (7.4 kcal/mol), ρ, and Kendall's τ. All MSE values from multiscale calculations are positive and identical to the MAD values, indicating that all methods overestimate the activation energies. The lowest MAD values belong to QM/MM-10.0/0.0 with electrostatic and mechanical embedding (6.2 and 7.6 kcal/mol, respectively), with QM1/QM2-7.5 calculations ranking third and fourth (8.7 and 10.4 kcal/mol for electrostatic and mechanical embedding, respectively). MADtr and MAXtr values are consistent for the same type of method, with QM1/QM2-4.5 with electrostatic and mechanical embedding ranking first and second, respectively. These methods have the closest range to the experimental range after QM/MM-7.5/2.5-Elec (Elec and Mech after the method names refer to the electrostatic and mechanical embedding, respectively).

**Table 1:**
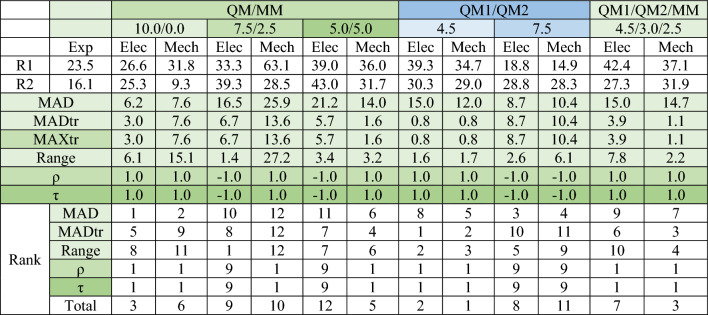
Experimental and calculated activation energies (kcal/mol) of S_N_2 reactions, with MAD, MADtr, MAXtr, Range, ρ, and τ values for each method, along with method ranks in corresponding quality measures and overall rank. Exp denotes the experimental values. R1 and R2 denote the reactions of NH_2_OH and NH_2_O^−^ with methyl iodide, respectively.

In the QM/MM block, the value before the slash indicates the thickness of the MM-active region, and the value after the slash denotes the thickness of the MM-fixed region in Å. The numbers below the QM1/QM2 block represent the thickness of the QM2 region around the QM1 region. For the QM1/QM2/MM calculations, the QM2 region has a thickness of 4.5 Å, while the MM active and MM fixed regions have thicknesses of 3.0 Å and 2.5 Å, respectively. Elec and Mech after the method names refer to the electrostatic and mechanical embedding, respectively. Overall ranks (last row) of the methods are based on MAD, MADtr, range, ρ, and τ ranks.* R*^2^ and MAXtr are not considered in determining the overall rank. This is because *R*^2^ is identical across all methods, and MAXtr corresponds directly with the MADtr values for each method category.

All *R*^2^ values are 1.0, which is expected for comparisons involving only two points, so we have not included them in Table 1. Additionally, all ρ and τ values are either 1.0 or − 1.0. Positive ρ and τ values correctly predict that the activation energy of the first S_N_2 reaction is higher than that of the second, while negative values indicate incorrect predictions. Among the methods used, QM/MM-7.5/2.5-Elec, QM/MM-5.0/5.0-Elec, and QM1/QM2-7.5 have inaccurately predicted the order of activation energy magnitudes for the S_N_2 reactions. Overall ranks of the methods, based on MAD, MADtr, range, ρ, and τ ranks (*R*^2^ and MAXtr are not considered in determining the overall rank. This is because *R*^2^ is identical across all methods, and MAXtr corresponds directly with the MADtr values for each method category), indicate that QM1/QM2-4.5 with electrostatic and mechanical embedding holds the top total rank, with QM1/QM2/MM ranking third (cf. the last row in Table 1).

From the results, conclusions can be drawn regarding the impact of the size of the MM-active and MM-fixed regions on the accuracy of computed activation energies. Among the employed QM/MM methods, QM/MM-10.0/0.0 exhibited the lowest MAD values. Notably, this variant with electrostatic embedding achieved the best overall rank (rank 3) among the QM/MM methods. This suggests that larger MM-active regions tend to yield activation energies closer to experimental values. Considering CPU time constraints, our results indicate that the CPU time for QM/MM calculations depends on the overall size of the MM-active and MM-fixed regions. Therefore, when the size of the QM region is fixed, it is advisable to choose a larger MM-active region, up to the hardware's capacity, and avoid including the MM-fixed region in the model. However, should covalent bonds cross the boundary of the MM-active region, it is advisable to incorporate additional atoms into the MM-fixed region to satisfy the valency of the impacted atoms (this is available in ORCA as is discussed in section "Multiscale Implementation in ORCA"). To address this issue, we developed the *pdbtoorca* program, which ensures that no covalent bond is cut at the MM-active boundary if it traverses solvent molecules. It includes an option that, if only one atom of a residue is included within the defined distance from the QM system, all atoms of that residue are incorporated into the MM-active region. However, cutting a covalent bond is unavoidable if the MM-active region intersects with protein residues rather than the solvent medium.

### Mechanism of the Claisen rearrangement

In this section, we delve into the results obtained from studying the mechanism of the Claisen rearrangement. We aim to ascertain whether the QM-only and multiscale methods can accurately reproduce the generally accepted reaction mechanism of Claisen rearrangements, as depicted in Fig. 3—a task for which QM-only calculations previously faltered for S_N_2 reactions, except when a constraint was applied to the NCI angle. To achieve this, we utilized the QM-only method, QM/MM with MM-active and -fixed region sizes of 4.0 and 2.5 Å, respectively, QM1/QM2 with a QM2 region radius around the QM1 region of 4.5 Å, and QM1/QM2/MM with QM2, MM-active, and MM-fixed region radii of 2.0, 2.5, and 2.5 Å, respectively. All methods were executed with two variants—electrostatic or mechanical embedding—yielding a total of seven methods (one QM-only and six multiscale methods).

Fig. 3 illustrates the mechanism of the Claisen rearrangement, wherein a single bond forms between C5 and C1, the double bond C1–C2 transforms into a single bond, the single bond C2–O becomes double, the C3–O bond is broken, the single bond C3–C4 transitions into a double bond, and the double bond C4–C5 converts into a single bond from the Re to the Pr state (the atoms are named on Fig. 3). Distances obtained from all multiscale methods closely aligned with those of the QM-only structures, with maximum MAD values of 0.29 Å (see the last rows of Tables S3-S8 in the Supporting Information). Average distances in the Re, TS, and Pr structures of the reaction are collated from all employed methods in Fig. 3. Additionally, Mayer bond orders for each bond are shown in the figure. Notably, the C3–O bond elongated from 1.45 to 2.12 Å from Re to TS, while the average C1–C5 bond length shortened from 2.29 to 1.55 Å from TS to Pr. These observations indicate that the O3–O bond is breaking and the C1–C5 bond is forming in the TS of the Claisen reaction, consistent with the generally accepted concerted mechanism of Claisen rearrangement. Calculated bond orders further support the proposed mechanism, with the C4–C5 bond order decreasing from 2.0 to 1.0, and the bond order of C2–O transitioning from 0.9 to 2.1 going from Re to Pr. In summary, both the QM-only and multiscale methods accurately reproduce the generally accepted mechanism for Claisen rearrangements.

### The activation free energy of the Claisen rearrangement

Finally, we analyze which of the employed methods is more accurate in reproducing the experimental activation free energies of the Claisen rearrangement. Although no directly reported activation energy is available for the Claisen rearrangement, experimental rate constants for this reaction in water, methanol, and three different mixtures of these solvents were determined (0.79, 1.60, 4.60, 11.00, and 18.00 × 10^5^ s^−1^ for the reaction in pure methanol (MET), 75% methanol (MET75%), 50% methanol (MET50%), 25% methanol (MET25%), and water (WAT), respectively^9^. We converted the rate constants to activation free energies using the general equation from transition state theory:

2 $$\Delta G^{\ddag } \, = \,\, - \,RT{\text{ln}}(kh / (k_{{\text{B}}} \, * \,T))$$

where *R* is the gas constant, *T* is the temperature at which the experimental rate constants are measured (333 K), *h* is Planck’s constant, *k*_B_ is Boltzmann's constant, and *k* is the experimental rate constant of the Claisen rearrangement in different solvents.

To expand the number of multiscale methods, we performed single-point calculations on the structures from the multiscale calculations with electrostatic embedding using mechanical embedding (Mech-Sp) and also optimizing the stationary structure along the reaction path with mechanical embedding (Mech-Opt), yielding three variants for each multiscale method and ten different methods overall. Computational activation free energies were calculated by adding zero-point energy (ZPE), thermal corrections, and entropy values to the corresponding multiscale energies. These values were obtained from frequency calculations performed on the optimized QM-only structures, as conducting frequency calculations on the entire QM/MM systems was not feasible due to hardware limitations..

The CPCM calculations yielded the same Δ*G*^‡^ value for all solvents (25.6 kcal/mol), indicating that the CPCM method is not suitable for calculating Δ*G*^‡^ of the Claisen rearrangement. The failure of the CPCM method in predicting the correct Δ*G*^‡^ values could be attributed to the similar polarity and volume of the Re and TS states of the Claisen rearrangement, which are almost identical due to the simple positional changes of some bonds (cf. Fig. 3). Consequently, we excluded this method from our dataset and focused solely on the multiscale methods.

Table 2 presents the experimental and calculated Δ*G*^‡^ values for the Claisen rearrangement along with the quality measures. The MAD values ranged between 5.0 and 11.0 kcal/mol, with the lowest MAD observed for QM1/QM2-Mech-Sp. Similar to the S_N_2 reactions, all multiscale methods produced positive MSE values, suggesting an overestimation of the Δ*G*^‡^ values.

**Table 2:**
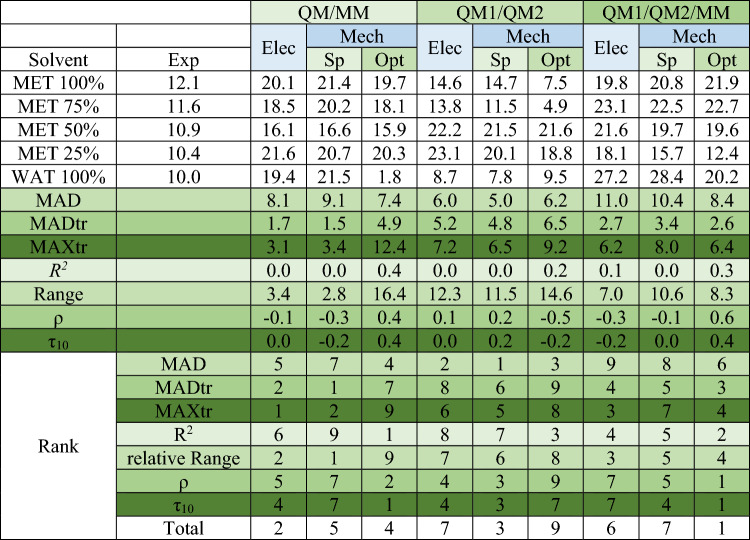
Experimental and calculated Δ*G*^‡^ values (kcal/mol) of the Claisen rearrangement, with MAD, MADtr, MAXtr, *R*^2^, Range, ρ, and τ_10_ (the τ_10_ values were calculated for all 10 pairs of the energies) values for each method, along with method ranks in corresponding quality measures and overall rank.

‘Elec’ and ‘Mech’ after the method names denote electrostatic and mechanical embedding, respectively. ‘Sp’ and ‘Opt’ indicate that single-point calculations were performed on the structures from the multiscale calculations with electrostatic embedding and that the stationary structure was optimized along the reaction path. Overall ranks (last row) of the methods are based on MAD, MADtr, MAXtr, *R*^2^, Range, ρ, and τ_10_ ranks.

The lowest MADtr and MAXtr values were recorded for QM/MM-Elec and QM/MM-Mech-Sp. While all *R*^2^ values were poor, the largest value belonged to QM/MM-Mech-Opt. Additionally, the lowest relative range was observed for QM/MM (Elec and Mech-Sp). Notably, the best ρ and τ_10_ (the τ_10_ values were calculated for all 10 pairs of the energies) values were obtained for QM1/QM2/MM. Overall, QM1/QM2/MM claimed the top rank (first place), with QM/MM-Elec securing the second rank.

The purpose of performing single-point calculations with mechanical embedding on structures optimized with electrostatic embedding was to examine the impact of maintaining consistency in the embedding scheme during both optimization and single-point calculations on the computed activation free energies. As shown in Table 2, the Δ*G*^‡^ values change after optimizing the structures with mechanical embedding. Interestingly, Mech-Opt exhibits a better overall rank than Mech-Sp in the QM/MM and QM1/QM2/MM calculations, as demonstrated in the last row of Table 2. Surprisingly, the best overall rank is achieved by the QM1/QM2/MM calculations with Mech-Opt, where both the optimization of the stationary structures and energy calculations are carried out with mechanical embedding. These observations suggest that maintaining consistency in the embedding scheme during optimization and energy calculations can lead to improved results.

**Figure 4:**
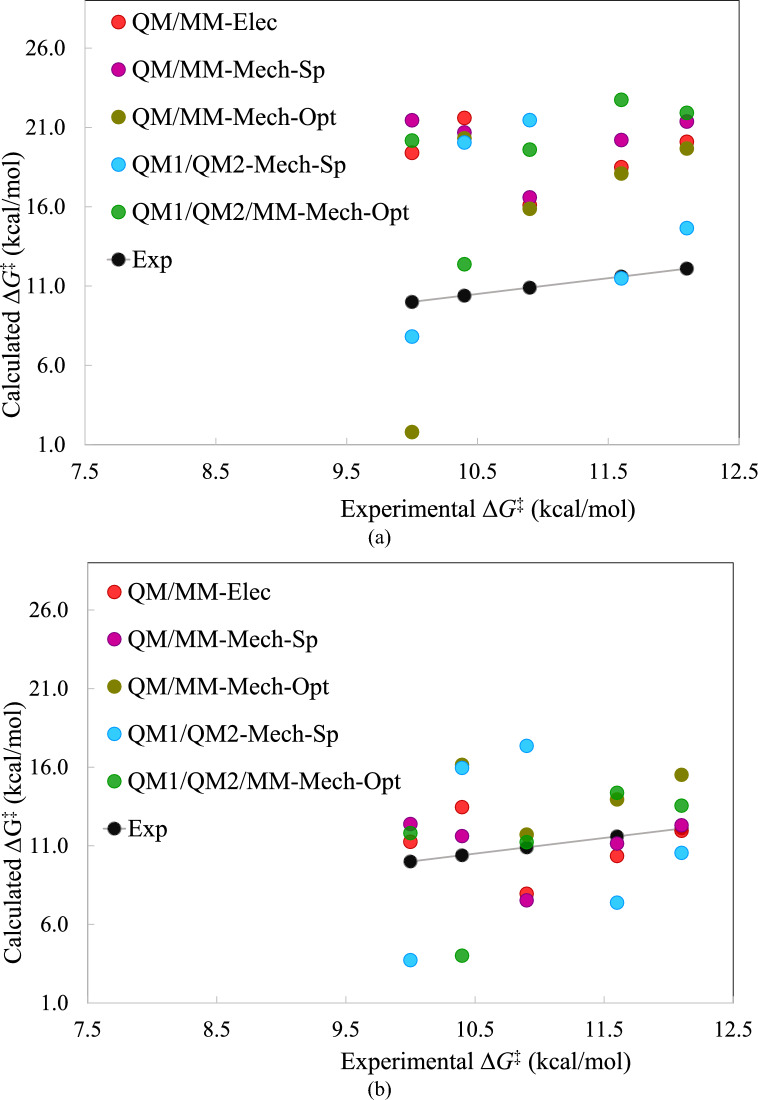
Performance of multiscale methods in studying the Claisen rearrangement for (a) absolute Δ*G*^‡^ and (b) the energies adjusted for systematic error (MSE).

Fig. 4 displays the efficacy of the five best methods: QM/MM-Elec, QM/MM-Mech-Sp, QM/MM-Mech-Opt, QM1/QM2-Mech-Sp, and QM1/QM2/MM-Mech-Opt. It is evident that all calculated absolute Δ*G*^‡^ values lie above the calibration line, indicating an overestimation of most values. The only exceptions are the Δ*G*^‡^ values of the reaction in pure water solvent from QM1/QM2-Mech-Sp and QM/MM-Mech-Opt (cf. Fig. 4a). From the methods, QM1/QM2-Mech-Sp yields values closest to the calibration line. Upon removing systematic errors (ranging from 1.3 to 11.0 kcal/mol), some points fall below the calibration line, resulting in a more symmetric distribution of calculated Δ*G*^‡^ values around the calibration line (cf. Fig. 4b). This implies that systematic errors are the primary source of deviation of the calculated values from the experimental ones. The precision of Eq. 2 could potentially contribute to these systematic errors, as it appears to underestimate experimental Δ*G*^‡^ values derived from the rate constants. After removing systematic errors, the Δ*G*^‡^ values of QM/MM-Mech-Sp and QM/MM-Mech-Opt become the closest to the calibration line.

## Conclusion

In this study, we employed QM and multiscale computational methods to investigate the reaction mechanisms and kinetics of the S_N_2 reactions of CH_3_I and either NH_2_OH or NH_2_O^−^, and the Claisen rearrangement of 8-(vinyloxy)dec-9-enoate. Our results offer insights into the performance and accuracy of these computational approaches in predicting experimental outcomes for organic reactions. Furthermore, we introduce a Python code to facilitate setting up multiscale calculations in ORCA, available on GitHub at https://github.com/iranimehdi/pdbtoORCA.

For the S_N_2 reactions, our results demonstrate the necessity of explicitly including solvent molecules in the calculations to accurately reproduce the transition state geometry and energetics. Multiscale methods, particularly QM/MM and QM1/QM2 showed promising performance in predicting activation energies, with some variants exhibiting close agreement with experimental values. Additionally, we observed that the size of the MM active region significantly influences the accuracy of calculated activation energies, emphasizing the importance of careful consideration when setting up multiscale simulations.

In the case of the Claisen rearrangement, both QM-only and multiscale methods successfully reproduced the proposed reaction mechanism. However, the activation free energies calculated using the continuum solvation model (CPCM), based on single-point calculations of QM-only structures, did not account for solvent effects. Conversely, multiscale methods more accurately reflected the solvents' impact on activation free energies, with QM1/QM2/MM-Mech-Opt and QM/MM-Elec ranking 1^st^ and 2^nd^ overall, respectively. Moreover, the accuracy of the results was enhanced upon the correction of systematic errors, as depicted in Fig. 4b.

Our study aimed to investigate the efficiency and accuracy of various computational methods in predicting reaction mechanisms and kinetics by testing multiple options for the radii defining each region (QM2, MM-active, and MM-fixed). While our results offer valuable insights, it is important to exercise caution when extrapolating these results to a broader range of reactions and systems. Future research should incorporate a more diverse set of reactions and larger systems to validate and refine the conclusions drawn from our current study. To further enhance the predictive capabilities of theoretical approaches in organic reactions, future research could explore novel multiscale strategies and refine computational models, ultimately contributing to the advancement of the field.

### Supporting information available

Further details about the ORCA program; A sample ORCA input file for performing QM/MM calculations; Geometrical parameters for the S_N_2 reactions from the employed methods; Geometrical parameters for the Claisen rearrangement from the employed methods; Graphs of experimental dielectric constants and densities of methanol/water mixture versus the mass percentage of methanol; Detailed GAFF parameters of the reactants; An input file of (*packmol.inp*) water/methanol solvent with weight percentages of 50/50% for the Packmol package.

### Supplementary Information

Supplementary Information.

## Data Availability

All data generated or analyzed during this study are included in this published article and its supplementary information file. Additionally, executables of the *pdbtoorca* program, a Python code for setting up multiscale calculations with ORCA, are available on GitHub at https://github.com/iranimehdi/pdbtoORCA.
