# Supplementary material for: Assessing the accuracy and efficacy of multiscale computational methods in predicting reaction mechanisms and kinetics of SN2 reactions and Claisen rearrangement
Source: Sci Rep. 2024 Jul 22;14:16791. doi: 10.1038/s41598-024-67468-x (PMC11263649; doi:10.1038/s41598-024-67468-x)
Supplement: Supplementary file 1 — Supplementary Information. [file 41598_2024_67468_MOESM1_ESM.pdf]

Assessing the Accuracy and Efficacy of Multiscale Computational Methods in Predicting Reaction Mechanisms and Kinetics of S_N2 Reactions and Claisen Rearrangement

*Maryam Haji Dehabadi, Hamid Saidi, Faezeh Zafari, and Mehdi Irani**

Department of Chemistry, University of Kurdistan, P.O.C 66177-15175, Sanandaj, Iran

Correspondence to Mehdi Irani, E-mail: m.irani@uok.ac.ir,

Tel: +98 – 9128018046

2024-04-24

https://doi.org/10.1038/s41598-024-67468-x

1. Further details about the ORCA program

The ORCA project originated in the mid-1990s as an internal initiative aimed at understanding specific characteristics in the spectroscopy of open-shell transition metal complexes.[1] Since then, it has matured into a versatile chemistry software suite, widely adopted by an academic user base exceeding 40,000 individuals. Citation metrics underscore ORCA's position as the second most widely used QM program currently available. ORCA distinguishes itself with several notable features, including state-of-the-art performance, a comprehensive theoretical framework, a user-friendly interface, and platform independence. These attributes collectively enable efficient computational studies spanning diverse domains of chemistry.

2. A sample ORCA input file for performing QM/MM calculations

```
# ORCA's input file for performing QM/MM calculations.
# This file is generated by a home-made Python program (pdbtoorca) by Mehdi and Maryam.
# The program is for free. However, cite it as follows, if you use it in your project.
# DOI: ................
# The working directory is: /home/zafari/met/test20
# The setup time and date are 18:44:16 and 2023-08-01, respectively.
! RIJCOSX TPSSh def2-SVP def2/J D3BJ TIGHTSCF Opt
!QMMM
%qmmm
ORCAFFFilename "prmtop.ORCAFF.prms"
# Excluding junctions, 34 atoms are included in the QM system, as follows.
QMatoms {0:33 } end
Use_QM_InfoFromPDB false
# The thickness of the active region is 4.0 angstroms (from the QM system). A total of 226 atoms are included in the active region, as follows.
ActiveAtoms {0:33 112:117 136:141 172:177 196:201 232:237 436:441 466:471 496:501 568:573 640:645 658:663 748:753 838:849 994:999 1036:1041 1270:1275 1462:1467 1498:1503 1516:1521 1594:1599 1606:1611 1618:1623 1636:1641 1738:1749 1798:1803 1810:1815 1834:1839 1846:1851 2062:2067 2170:2175} end
Use_Active_InfoFromPDB false
# An extension shell of 2.5-angstrom thickness will surround the active region.
# There are 2446 atoms in your PDB file, from which 226 atoms are included in the active region and 240 atoms are included in the extension shell. Hence, 1980 atoms will be ignored in the calculations.
OptRegion_FixedAtoms {52:57 64:69 76:87 94:99 214:219 238:243 274:279 364:369 454:459 520:525 598:603 718:723 790:795 814:819 892:897 1000:1011 1120:1125 1180:1185 1246:1251 1300:1305 1384:1389 1408:1413 1432:1437 1450:1455 1492:1497 1774:1779 1942:1959 2008:2013 2044:2049 2056:2061 2176:2181 2230:2235 2254:2259 2278:2283 2326:2331 2404:2409} end
end
% geom scan
B 0 10 = 4.3, 1.4, 19
end
end
% pal
nprocs
4
end
*pdbfile -1 1 orcapdb.pdb
```

3. Geometrical parameters for the S_N2 reactions from the employed methods

Table S1. Geometrical parameters obtained from the multiscale methods employed in studying the reaction between NH_2OH and methyl iodide for the Re, TS, and Pr states. Distances are expressed in Å, and the NCI angles are in degrees. The optimization procedure is performed with electrostatic embedding. The row below the QM/MM block represents the thickness of the MM-active region around the QM region (the number before the slash) and the thickness of the MM-fixed region around the MM active region (the number after the slash). The numbers below the QM1/QM2 block represent the thickness of the QM2 region around the QM1 region. For the QM1/QM2/MM calculations, the thicknesses of the QM2, MM active, and MM fixed regions are set at 4.5, 3.0, and 2.5, respectively. All the values for the thickness of the QM2, MM-active, and MM-fixed regions are in Å.

	QM/MM									QM1/QM2						QM1/QM2/MM		
	10.00/0.0			7.5/2.5			5.0/5.0			4.5			7.5					
	Re	TS	Pr	Re	TS	Pr	Re	TS	Pr	Re	TS	Pr	Re	TS	Pr	Re	TS	Pr
O-N	1.43	1.44	1.40	1.43	1.46	1.40	1.43	1.47	1.41	1.43	1.48	1.42	1.44	1.45	1.40	1.42	1.42	1.40
N-C		2.11	1.47		2.20	1.50		2.17	1.50		2.10	1.50		2.10	1.50		1.90	1.70
C-I	2.17	2.44		2.16	2.28		2.16	2.29		2.14	2.18		2.18	2.35		2.14	2.82	
<NCI		167.3			177.8			177.9			154.7			165.7			176.2	

Table S2. Geometrical parameters obtained from the multiscale methods employed in studying the reaction between NH_2O^- and methyl iodide for the Re, TS, and Pr states. Distances are expressed in Å, and the NCI angles are in degrees. The optimization procedure is performed with electrostatic embedding. The row below the QM/MM block represents the thickness of the MM active region around the QM region (the number before the slash) and the thickness of the MM fixed region around the MM active region (the number after the slash). The numbers below the QM1/QM2 block represent the thickness of the QM2 region around the QM1 region. For the QM1/QM2/MM calculations, the thicknesses of the QM2, MM active, and MM fixed regions are set at 4.5, 3.0, and 2.5, respectively. All the values for the thickness of the QM2, MM-active, and MM-fixed regions are in Å.

	QM/MM									QM1/QM2						QM1/QM2/MM		
	10.00/0.0			7.5/2.5			5.0/5.0			4.5			7.5					
	Re	TS	Pr	Re	TS	Pr	Re	TS	Pr	Re	TS	Pr	Re	TS	Pr	Re	TS	Pr
O-N	1.45	1.44	1.39	1.44	1.46	1.38	1.44	1.46	1.38	1.41	1.39	1.38	1.41	1.40	1.38	1.44	1.44	1.39
N-C		2.25	1.49		2.22	1.49		2.21	1.50		2.30	1.50		2.50	1.50		2.30	1.50
C-I	2.16	2.35		2.17	2.28		2.17	2.28		2.17	2.27		2.17	2.23		2.16	2.27	
<NCI		165.3			163.2			163.5			168.7			152.9			167.4	

4. Geometrical parameters for the Claisen rearrangement from the employed methods

Table S3. Key distances (in Å) obtained from QM-only and the QM/MM method with electrostatic embedding used to study the Claisen rearrangement for the Re, TS, and Pr states. The last row represents MAD values with respect to the QM-only structure.

				QM/MM														
	QM-only			Water			25%Met			50%Met			75%Met			Met		
	Re	TS	Pr	Re	TS	Pr	Re	TS	Pr	Re	TS	Pr	Re	TS	Pr	Re	TS	Pr
C1-C5		2.27	1.54		2.35	1.55		2.20	1.56		2.33	1.56		2.33	1.54		2.21	1.54
C3-O	1.43	2.55		1.45	2.45		1.45	2.45		1.45	2.39		1.45	2.46		1.45	2.45	
C4-C5	1.34	1.36	1.50	1.34	1.34	1.50	1.34	1.34	1.50	1.34	1.34	1.50	1.34	1.34	1.51	1.34	1.34	1.50
C3-C4	1.51	1.47	1.34	1.50	1.50	1.34	1.50	1.50	1.34	1.50	1.50	1.34	1.50	1.50	1.34	1.51	1.51	1.34
C2-O	1.35	1.33	1.21	1.37	1.37	1.22	1.36	1.36	1.22	1.36	1.36	1.22	1.36	1.35	1.22	1.36	1.36	1.21
C1-C2	1.34	1.35	1.51	1.34	1.34	1.50	1.34	1.34	1.51	1.34	1.34	1.50	1.34	1.35	1.50	1.34	1.34	1.51
MAD	0.00	0.00	0.00	0.01	0.05	0.01	0.01	0.04	0.01	0.01	0.05	0.01	0.01	0.04	0.01	0.01	0.04	0.00

Table S4. Key distances (in Å) obtained from the QM1/QM2 method with electrostatic embedding used to study the Claisen rearrangement for the Re, TS, and Pr states. The final row indicates MAD relative to the QM-only structure.

	QM1/QM2														
	Water			25%Met			50%Met			75%Met			Met		
	Re	TS	Pr	Re	TS	Pr	Re	TS	Pr	Re	TS	Pr	Re	TS	Pr
C1-C5		2.44	1.56		2.20	1.54		2.33	1.56		2.64	1.57		2.21	1.54
C3-O	1.46	2.54		1.45	2.45		1.45	2.45		1.46	1.50		1.45	2.45	
C4-C5	1.33	1.35	1.49	1.34	1.34	1.50	1.34	1.34	1.50	1.34	1.34	1.50	1.34	1.35	1.51
C3-C4	1.50	1.48	1.34	1.50	1.50	1.35	1.50	1.50	1.35	1.50	1.49	1.34	1.50	1.51	1.35
C2-O	1.37	1.37	1.22	1.35	1.35	1.22	1.36	1.36	1.22	1.34	1.35	1.22	1.35	1.35	1.22
C1-C2	1.34	1.34	1.49	1.34	1.34	1.50	1.34	1.34	1.50	1.35	1.34	1.49	1.35	1.35	1.51
MAD	0.01	0.04	0.01	0.01	0.04	0.01	0.01	0.04	0.01	0.01	0.25	0.01	0.01	0.04	0.01

Table S5. Key distances (in Å) obtained from the QM1/QM2/MM method with electrostatic embedding used to study the Claisen rearrangement for the Re, TS, and Pr states. The final row indicates MAD relative to the QM-only structure.

	QM1/QM2/MM														
	Water			25%Met			50%Met			75%Met			Met		
	Re	TS	Pr	Re	TS	Pr	Re	TS	Pr	Re	TS	Pr	Re	TS	Pr
C1-C5		2.20	1.54		2.39	1.57		2.28	1.57		1.81	1.54		2.21	1.55
C3-O	1.44	2.44		1.45	2.45		1.45	2.45		1.45	3.36		1.45	2.45	
C4-C5	1.34	1.34	1.50	1.34	1.34	1.50	1.34	1.34	1.50	1.34	1.48	1.50	1.34	1.34	1.51
C3-C4	1.50	1.50	1.34	1.50	1.50	1.34	1.50	1.50	1.34	1.50	1.34	1.34	1.50	1.50	1.34
C2-O	1.36	1.36	1.21	1.36	1.36	1.21	1.36	1.36	1.21	1.36	1.22	1.21	1.35	1.36	1.21
C1-C2	1.34	1.34	1.50	1.34	1.34	1.51	1.34	1.34	1.51	1.35	1.48	1.50	1.34	1.34	1.51
MAD	0.00	0.05	0.00	0.01	0.05	0.01	0.01	0.03	0.01	0.01	0.29	0.00	0.01	0.04	0.00

Table S6. Key distances (in Å) obtained from QM-only and the QM/MM method with mechanical embedding used to study the Claisen rearrangement for the Re, TS, and Pr states. The final row indicates MAD relative to the QM-only structure.

				QM/MM														
	QM-only			Water			25%Met			50%Met			75%Met			Met		
	Re	TS	Pr	Re	TS	Pr	Re	TS	Pr	Re	TS	Pr	Re	TS	Pr	Re	TS	Pr
C1-C5		2.27	1.54		2.35	1.55		2.19	1.55		2.32	1.55		2.32	1.54		2.20	1.53
C3-O	1.43	2.55		1.44	1.55		1.44	1.60		1.45	1.53		1.45	1.55		1.45	1.61	
C4-C5	1.34	1.36	1.50	1.33	1.34	1.50	1.33	1.37	1.50	1.33	1.35	1.50	1.33	1.35	1.50	1.33	1.37	1.50
C3-C4	1.51	1.47	1.34	1.5	1.47	1.34	1.50	1.46	1.34	1.50	1.48	1.34	1.50	1.48	1.34	1.51	1.46	1.34
C2-O	1.35	1.33	1.21	1.35	1.35	1.21	1.35	1.32	1.21	1.35	1.34	1.21	1.35	1.34	1.21	1.35	1.32	1.21
C1-C2	1.34	1.35	1.51	1.34	1.35	1.50	1.34	1.37	1.52	1.34	1.35	1.51	1.34	1.35	1.51	1.34	1.37	1.52
MAD	0.00	0.00	0.00	0.00	0.19	0.00	0.00	0.18	0.01	0.01	0.18	0.00	0.01	0.18	0.00	0.01	0.18	0.01

Table S7. Key distances (in Å) obtained from the QM1/QM2 method with mechanical embedding used to study the Claisen rearrangement for the Re, TS, and Pr states. The final row indicates MAD relative to the QM-only structure.

	QM1/QM2														
	Water			25%Met			50%Met			75%Met			Met		
	Re	TS	Pr	Re	TS	Pr	Re	TS	Pr	Re	TS	Pr	Re	TS	Pr
C1-C5		2.26	1.56		2.36	1.54		2.32	1.56		2.32	1.56		2.36	1.53
C3-O	1.46	2.49		1.45	1.55		1.45	1.57		1.45	1.58		1.45	1.54	
C4-C5	1.33	1.41	1.49	1.33	1.34	1.50	1.33	1.35	1.50	1.33	1.35	1.49	1.33	1.35	1.50
C3-C4	1.50	1.37	1.34	1.50	1.47	1.34	1.50	1.47	1.34	1.50	1.47	1.34	1.50	1.48	1.34
C2-O	1.37	1.26	1.22	1.36	1.35	1.21	1.36	1.34	1.21	1.35	1.35	1.21	1.36	1.34	1.21
C1-C2	1.34	1.41	1.48	1.34	1.35	1.50	1.34	1.35	1.49	1.35	1.35	1.49	1.34	1.35	1.51
MAD	0.01	0.06	0.01	0.01	0.19	0.00	0.01	0.18	0.01	0.01	0.18	0.01	0.01	0.19	0.00

Table S8. Key distances (in Å) obtained from the QM1/QM2/MM method with mechanical embedding used to study the Claisen rearrangement for the Re, TS, and Pr states. The final row indicates MAD relative to the QM-only structure.

	QM1/QM2/MM														
	Water			25%Met			50%Met			75%Met			Met		
	Re	TS	Pr	Re	TS	Pr	Re	TS	Pr	Re	TS	Pr	Re	TS	Pr
C1-C5		2.26	1.54		2.38	1.56		2.27	1.56		2.22	1.54		2.20	1.54
C3-O	1.44	1.56		1.44	1.53		1.44	1.57		1.45	1.63		1.44	1.64	
C4-C5	1.33	1.35	1.49	1.33	1.35	1.50	1.33	1.36	1.50	1.33	1.36	1.50	1.33	1.36	1.50
C3-C4	1.50	1.47	1.34	1.50	1.48	1.34	1.50	1.47	1.34	1.50	1.46	1.34	1.50	1.45	1.34
C2-O	1.35	1.33	1.20	1.36	1.35	1.20	1.36	1.34	1.20	1.36	1.32	1.21	1.35	1.31	1.20
C1-C2	1.34	1.35	1.50	1.34	1.35	1.51	1.34	1.36	1.51	1.34	1.36	1.50	1.34	1.37	1.51
MAD	0.00	0.17	0.01	0.01	0.20	0.01	0.01	0.17	0.01	0.01	0.17	0.00	0.00	0.17	0.00

5. Graphs of experimental dielectric constants and densities of methanol/water mixture versus the mass percentage of methanol

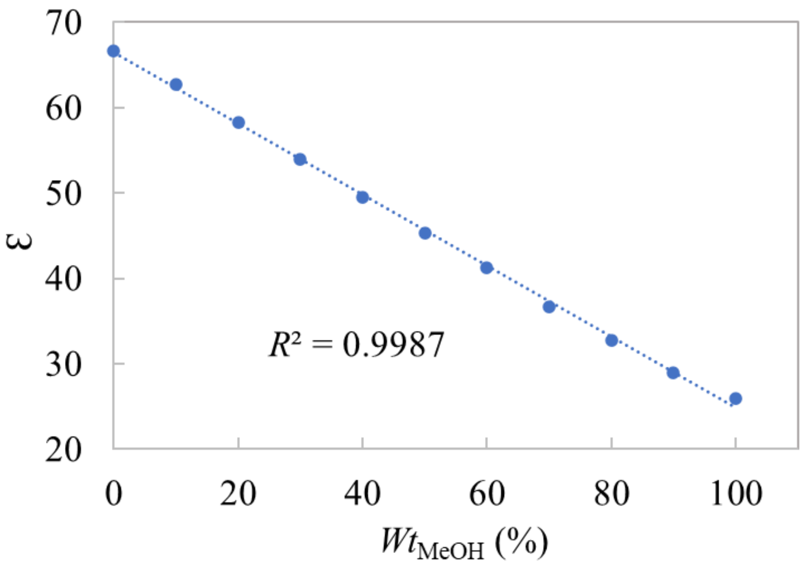

Figure S1. Dielectric constants (ε) versus methanol mass percentage for methanol/water mixtures. The experimental data were obtained from the work of Gösta Åkerlöf at 60 ºC.[2]

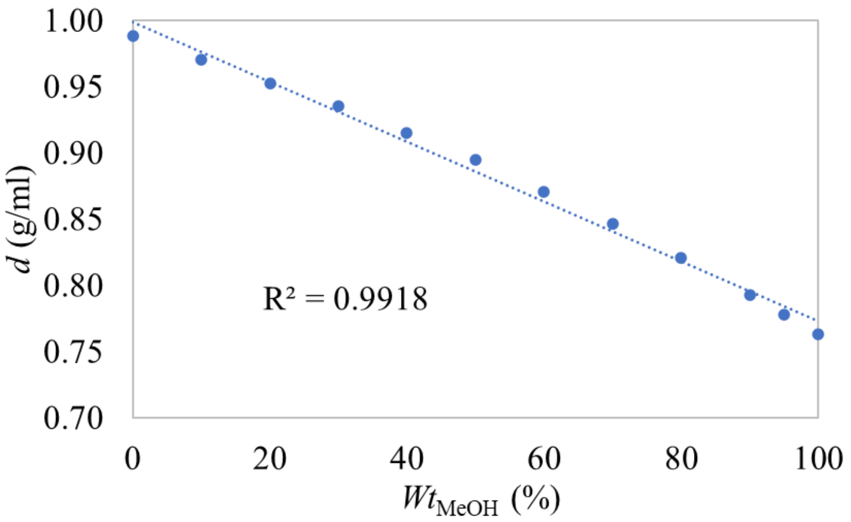

Figure S2. Density versus methanol mass percentage for methanol/water mixtures. The experimental data were obtained from the work of S. Z. Mikhail and W. R. Kimel.[3]

6. Detailed GAFF parameters of the reactants

6.1 GAFF parameters for hydroxylamine (NH_2OH)

```
This is a remark line
molecule.res
HAM   INT  0
CORRECT     OMIT DU   BEG
  0.0000
   1  DUMM  DU    M    0  -1  -2     0.000      .0        .0      .00000
   2  DUMM  DU    M    1   0  -1     1.449      .0        .0      .00000
   3  DUMM  DU    M    2   1   0     1.523   111.21       .0      .00000
   4  N1    n3    M    3   2   1     1.540   111.208  -180.000 -0.765002
   5  H1    hn    E    4   3   2     1.022   109.845   -32.599  0.401944
   6  H2    hn    E    4   3   2     1.022   109.839  -147.483  0.401944
   7  O1    oh    M    4   3   2     1.448    11.876   -90.000 -0.495460
   8  H3    ho    E    7   4   3     0.970   101.834     0.060  0.456574


LOOP

IMPROPER

DONE
STOP
```

6.2 GAFF parameters for methyl iodide (MEI)

```
This is a remark line
molecule.res
MEI   INT  0
CORRECT     OMIT DU   BEG
  0.0000
   1  DUMM  DU    M    0  -1  -2     0.000      .0        .0      .00000
   2  DUMM  DU    M    1   0  -1     1.449      .0        .0      .00000
   3  DUMM  DU    M    2   1   0     1.523   111.21       .0      .00000
   4  C1    c3    M    3   2   1     1.540   111.208  -180.000 -0.606799
   5  H1    h1    E    4   3   2     1.088   107.320   124.134  0.237272
   6  H2    h1    E    4   3   2     1.087   107.334  -115.874  0.237272
   7  H3    h1    E    4   3   2     1.088   107.324     4.141  0.237272
   8  I1    i     M    4   3   2     2.187     0.000   -90.000 -0.105016


LOOP

IMPROPER

DONE
STOP
```

6.3 GAFF parameters for amine oxide anion (NH_2O^-)

```
This is a remark line
molecule.res
AMO   INT  0
CORRECT     OMIT DU   BEG
  0.0000
   1  DUMM  DU    M    0  -1  -2     0.000      .0       .0     .00000
   2  DUMM  DU    M    1   0  -1     1.449      .0       .0     .00000
   3  DUMM  DU    M    2   1   0     1.523   111.21      .0     .00000
   4  N1    n3    M    3   2   1     1.540   111.208  -180.000 -0.712723
   5  H1    hn    E    4   3   2     1.066   113.197   -35.490  0.208064
   6  H2    hn    E    4   3   2     1.066   113.197  -144.510  0.208064
   7  O1    o     M    4   3   2     1.409     7.544   -90.000 -0.703406


LOOP

IMPROPER

DONE
STOP
```

6.4 GAFF parameters for 8-(vinyloxy)dec-9-enoate

```
This is a remark line
molecule.res
AEN   INT  0
CORRECT     OMIT DU   BEG
  0.0000
   1  DUMM  DU    M    0  -1  -2     0.000      .0        .0      .00000
   2  DUMM  DU    M    1   0  -1     1.449      .0        .0      .00000
   3  DUMM  DU    M    2   1   0     1.523   111.21       .0      .00000
   4  C1    c2    M    3   2   1     1.540   111.208  -180.000 -0.635805
   5  H1    ha    E    4   3   2     1.084   139.375   -49.020  0.220665
   6  H2    ha    E    4   3   2     1.084    94.286   165.424  0.220665
   7  C2    c2    M    4   3   2     1.335    35.044    25.830  0.088235
   8  H3    h4    E    7   4   3     1.091   121.911    47.157  0.140018
   9  O1    os    M    7   4   3     1.358   122.851  -134.488 -0.269663
  10  C3    c3    M    9   7   4     1.439   116.613   168.301  0.329102
  11  H4    h1    E   10   9   7     1.103   108.326    37.055  0.030768
  12  C4    c2    B   10   9   7     1.505   108.403   154.432 -0.217616
  13  H5    ha    E   12  10   9     1.089   114.243  -178.520  0.146086
  14  C5    c2    B   12  10   9     1.332   125.735     1.615 -0.390546
  15  H6    ha    E   14  12  10     1.087   121.049   179.624  0.168788
  16  H7    ha    E   14  12  10     1.085   121.507    -0.435  0.168788
  17  C6    c3    M   10   9   7     1.541   110.096   -81.148 -0.269856
  18  H8    hc    E   17  10   9     1.098   108.204   -62.055  0.086083
  19  H9    hc    E   17  10   9     1.099   107.350    52.659  0.086083
  20  C7    c3    M   17  10   9     1.535   114.558   173.912  0.005898
  21  H10   hc    E   20  17  10     1.100   110.040    65.219  0.046396
  22  H11   hc    E   20  17  10     1.097   109.644   -51.165  0.046396
  23  C8    c3    M   20  17  10     1.536   112.890  -173.186 -0.081685
  24  H12   hc    E   23  20  17     1.101   108.879    58.730  0.016615
  25  H13   hc    E   23  20  17     1.102   108.703   -56.390  0.016615
  26  C9    c3    M   23  20  17     1.539   113.955  -179.208 -0.088914
  27  H14   hc    E   26  23  20     1.101   108.992    58.620  0.010237
  28  H15   hc    E   26  23  20     1.101   108.274   173.101  0.010237
  29  C10   c3    M   26  23  20     1.536   114.756   -64.424  0.159787
  30  H16   hc    E   29  26  23     1.096   111.724    60.706 -0.027162
  31  H17   hc    E   29  26  23     1.102   108.831   -55.576 -0.027162
  32  C11   c3    M   29  26  23     1.529   114.950  -178.662 -0.214286
  33  H18   hc    E   32  29  26     1.104   108.988   -48.481  0.010802
  34  H19   hc    E   32  29  26     1.099   111.646    68.444  0.010802
  35  C12   c     M   32  29  26     1.581   113.343  -167.880  0.841445
  36  O3    o     E   35  32  29     1.259   115.172    28.700 -0.818909
  37  O2    o     M   35  32  29     1.254   115.023  -152.995 -0.818909

LOOP
IMPROPER
   C2   H1   C1   H2
   C1   H3   C2   O1
   C5   C3   C4   H5
   C4   H6   C5   H7
  C11   O3  C12   O2
DONE
STOP
```

7. An input file of (*packmol.inp*) water/methanol solvent with weight percentages of 50/50% for the Packmol package

```
# This determines the minimum distance between molecules. The default value is 2 angstroms.
tolerance 2.0
# Specify the file format (PDB).
filetype pdb
# Output file name
output r1-Methanol.pdb
# Assign the solute file.
structure AEN.pdb
# Assign a number to the solute molecule (1 in this instance).
  number 1
# Place the solution's center of mass in the center of the box.
# These values relate to the -x, -y, -z, x, y, and z values of the center of mass.
# Alternatively, you may change them.
  fixed 15. 15. 15. 0. 0. 0.
  centerofmass
end structure
# add first type of solvent molecules
structure MET.pdb
  number 224
  inside cube 0. 0. 0. 30
end structure

# add second type of solvent molecules
structure WAT.pdb
  number 402
  inside cube 0. 0. 0. 30
end structure# Identify the cubic box's coordinates (-x, -y, -z, x, y, and z values).
  inside box -15. -15. -15. 15. 15. 15.
end structure
```

8. References

(1) Neese, F. *Electronic Structure and Spectroscopy of Novel Copper Chromophores in Biology*; UFO, Atelier für Gestaltung und Verlag, 1997.

(2) Åkerlöf, G. Dielectric Constants of Some Organic Solvent-Water Mixtures at Various Temperatures. *J. Am. Chem. Soc.* **1932**, *54* (11), 4125–4139. https://doi.org/10.1021/ja01350a001.

(3) Mikhail, S. Z.; Kimel, W. R. Densities and Viscosities of Methanol-Water Mixtures. *J. Chem. Eng. Data* **1961**, *6* (4), 533–537. https://doi.org/10.1021/je60011a015.
